# The impact of action video game experience on visual selective attention in deaf middle school students

**DOI:** 10.3389/fpsyg.2025.1633957

**Published:** 2025-10-01

**Authors:** Ting Cui, Dongmao Ye, Yifan Dong, Huina Gong, Yuanbing Guo, Zhi Gao

**Affiliations:** ^1^School of Sports Medicine, Wuhan Sports University, Wuhan, China; ^2^Department of Physical Education, Wuhan Textile University, Wuhan, China; ^3^School of Education, Central China Normal University, Wuhan, China; ^4^College of Sports Science and Technology, Wuhan Sports University, Wuhan, China; ^5^School of Physical Education, Wuhan Sports University, Wuhan, China

**Keywords:** deaf students, action video game experience, selective attention, eye-tracking, Perceptual Load Theory

## Abstract

**Introduction:**

Previous studies have shown that congenital deafness enhances peripheral visual processing but may reduce attention to central stimuli. In contrast, action video game experience has been shown to improve top-down attentional control and resistance to distraction. However, it remains unclear whether action video game experience can modify the peripheral attention bias typically observed in deaf individuals, particularly within the context of perceptual load theory. This question is crucial for understanding adaptive mechanisms and informing attention training interventions in the deaf population.

**Methods:**

To investigate the impact of action video game experience on visual selective attention in deaf middle school students, a response competition paradigm combined with eye-tracking technology was employed to systematically evaluate and compare the selective attention characteristics of four groups: deaf action video game players (Deaf VGPs), deaf non-video game players (Deaf NVGPs), hearing action video game players (Hearing VGPs), and hearing non-video game players (Hearing NVGPs). The comparison was conducted under varying levels of perceptual load and types of distractors.

**Results:**

(1) Deaf students generally demonstrated lower accuracy, slower reaction times, longer fixation durations, and more total fixation counts than their hearing counterparts in visual selective attention tasks. (2) Deaf VGPs performed better than deaf NVGPs, particularly in accuracy, and their performance in both accuracy and total fixation counts was comparable to that of their hearing counterparts, suggesting an association between action video game experience and enhanced selective attention in deaf students. (3) Deaf students, particularly NVGPs, exhibited larger compatible effects under both low and high perceptual load conditions, with the effect being more substantial under high load, indicating that perceptual load may exert a greater influence on deaf NVGPs.

**Conclusion:**

The mechanisms underlying selective attention processing in deaf students appear to be influenced by factors such as action video game experience and auditory deprivation-induced plasticity changes.

## 1 Introduction

There is an ongoing scholarly debate regarding the locus of selective attention, with two competing perspectives: early selection (pertaining to perceptual processing) and late selection (pertaining to response selection). The Perceptual Load Theory (PLT) has been proposed as a more robust framework for addressing this controversy. PLT explains the locus of attention by examining how perceptual load influenced the distractor compatible effect. The distractor compatible effect refers to differences in performance when distractors are either compatible or incompatible with the target. Under compatible conditions, responses may be facilitated (e.g., shorter reaction times), although interference can also occur due to feature suppression. Under incompatible conditions, distractors typically induce response competition, leading to impaired performance(e.g., longer reaction times) (Lavie, 1995). According to PLT, the locus of selective attention is contingent upon the perceptual load of the task. When the perceptual load is low and does not exceed cognitive capacity, distractors are processed, giving rise to compatible effects, aligning with the late selection perspective. Conversely, when the perceptual load is high, irrelevant distractors are not processed, and compatible effects disappear, supporting the early selection perspective (Lavie, 1995, 2005). Furthermore, studies that have manipulated perceptual load levels have demonstrated that two factors—congenital deafness and experience with action video games—can influence selective attention mechanisms (Lavie, 2005). The interplay between these two factors in shaping selective attention among deaf populations within the context of PLT remains an underexplored area of research.

The absence of auditory experience can significantly influence the development of visual abilities and induce alterations in cross-modal plasticity (Dye and Terhune-Cotter, 2023). Two primary perspectives exist regarding the nature of visual attention in deaf populations: the deficit perspective and the compensation perspective. The deficit perspective (Quittner et al., 1994; Smith et al., 1998) posits that auditory deprivation leads to impairments within the visual system, thereby diminishing visual attention in deaf individuals. Conversely, the compensation perspective (Neville and Lawson, 1987; Lore and Song, 1991) contends that functional reorganization of the visual system, driven by environmental demands, enhances visual attention in deaf individuals. An integrative framework suggests that the distinction between these two theories lies in their focus on different dimensions of visual attention in deaf individuals. Specifically, the deficit perspective emphasizes temporal allocation of attention, whereas the compensation perspective highlights spatial allocation of attention (Proksch and Bavelier, 2002; Dye and Bavelier, 2010). Empirical evidence demonstrates that deaf individuals exhibit superior performance in detecting visual targets in the peripheral visual field compared to hearing individuals. Their attentional resources are predominantly allocated to the periphery rather than the central visual field, which increases their sensitivity to peripheral stimuli. However, this heightened sensitivity also renders them more susceptible to distractions from irrelevant stimuli, potentially leading to reduced performance on tasks requiring sustained attention (Dye et al., 2009). Behavioral studies investigating selective attention in deaf populations reveal that their visual selective attention capacity is neither uniformly deficient nor fully compensated for, but varies across different regions of the visual field. Compared to hearing individuals, deaf individuals have been shown to demonstrate diminished attentional performance in the central visual field but enhanced performance in the peripheral visual field (Dye and Bavelier, 2010; Dye and Terhune-Cotter, 2023). For example, using an adapted perceptual-load paradigm, Proksch and Bavelier (2002) found that, compared with hearing controls, deaf participants exhibited longer reaction times across conditions. Moreover, as perceptual load increased, deaf participants showed smaller compatibility effects for central distractors but larger compatibility effects for peripheral distractors. Similar findings were also reported by Chen et al. (2010).

Action video games necessitate substantial visual and attentional resources. They share a common set of qualitative features, including exceptional speed, a high degree of perceptual, cognitive, and motor load, temporal and spatial unpredictability, and an emphasis on peripheral visual processing (Green et al., 2010). Research has demonstrated that experience with action video games is strongly associated with enhancements in various cognitive functions, such as perception, spatial cognition, and top-down attentional control (Bavelier and Green, 2019, 2024; Bediou et al., 2018). Notably, numerous empirical studies have employed action video game training to improve various aspects of attention across diverse populations, including healthy adults (Zhang et al., 2021; Argilés et al., 2023), the elderly (Anguera et al., 2013; Wong et al., 2022), children with dyslexia (Bertoni et al., 2024; Puccio et al., 2024), and patients with brain injuries (Azizi et al., 2022). Findings consistently demonstrate that engaging in action video games augments attentional control. Behavioral mechanism studies indicate that action video games improve selective attention performance among video game players (VGPs) by diminishing the influence of irrelevant distractors (Chisholm et al., 2010) and enhancing visual attention capacity (Green and Bavelier, 2003; Green et al., 2012). Compared to non-video game players (NVGPs), video game players (VGPs) exhibit an expanded spatial attentional span and demonstrate a greater compatibility effect as perceptual load increases (Green and Bavelier, 2003, 2015). Investigations into neural mechanisms reveal that action video games induce significant structural and functional alterations in multiple brain regions, including the prefrontal, parietal, and temporal cortices, as well as the hippocampus and striatum (Brilliant et al., 2019; Choi et al., 2021). Moreover, research on attentional enhancement in action video game players found that the mechanisms underlying attentional allocation and processing efficiency were altered. Specifically, compared to non-players, as perceptual load increased, gamers exhibited reduced recruitment of the fronto-parietal attentional network. This pattern suggested that players were more capable of filtering out irrelevant information at an early processing stage (Bavelier et al., 2012). Furthermore, these games enhance cognitive functions, such as perception, attention, and memory, by modulating neuroelectric activity (Anguera et al., 2013; Hilla et al., 2020).

Perceptual load plays a crucial role in shaping selective attention, and fixation-related parameters (including fixation duration and fixation counts) can serve as effective indicators of perceptual load (Liu et al., 2022; Harris et al., 2023). However, previous studies have reported inconsistent findings. For instance, as perceptual load increased, the fixation duration was found to have either increased (Harris et al., 2023) or decreased, while the fixation counts were observed to increase (Liu et al., 2022; Bend and Öörni, 2025). This divergence highlights the need to clarify the mechanisms underlying these effects. Conceptually, fixation duration primarily reflects the depth of attentional processing, whereas fixation counts provide critical information regarding the spatial allocation and shifting of attention. Taken together, these two indices offer a more comprehensive assessment of attentional processes (Henderson, 2017). Therefore, further research is needed to clarify the specific effects of perceptual load on fixation behavior. Eye-tracking methodologies facilitate the visualization of attentional resource allocation and its dynamic processes by recording an individual’s fixation points, saccadic trajectories, and pupil size, among other metrics (Souto and Kerzel, 2021). Individuals with profound deafness often rely more heavily on visual information to navigate their environment, resulting in notable changes in the function of their oculomotor brain networks, which subsequently affect their eye movement patterns (Bottari et al., 2012; Bélanger and Rayner, 2015). Action video games, recognized as effective cognitive training tools, have been associated with changes in eye movement behavior (Azizi et al., 2017; Montolio-Vila et al., 2024). Action video game players exhibited different eye fixation patterns during tasks, manifesting as shorter fixation durations and fewer fixations (Jeong et al., 2022; Li et al., 2022). Previous research on the attention characteristics of deaf individuals, as well as the impact of video games on their attention, has predominantly focused on behavioral dimensions (for example, Buckley et al., 2010; Nagendra et al., 2017; Holmer et al., 2020). Studies examining their eye movement characteristics have generally centered on reading and social communication contexts (for example, Bélanger and Rayner, 2015; Agrawal and Peiris, 2021).

In summary, further investigation is warranted to elucidate the mechanisms by which auditory deficits and action video game experience interact to modulate the processing of selective attention. This study employs the response competition paradigm within the Perceptual Load Theory (PLT) framework to systematically examine the selective attention profiles of four distinct groups: deaf action video game players (Deaf VGPs), deaf non-video game players (Deaf NVGPs), hearing action video game players (Hearing VGPs), and hearing non-video game players (Hearing NVGPs). The research will evaluate these groups under varying levels of perceptual load and types of distractors while analyzing eye-tracking data to explore the impact of action video game exposure on visual selective attention among deaf students. Grounded in prior evidence on the selective-attention profile of deaf individuals and on how perceptual load shapes distractor processing in both deaf populations and action video game players, we advanced two directional hypotheses. First, deaf middle school students are expected to exhibit longer reaction times, longer fixation durations, and more fixation counts during the selective attentional task compared to their hearing counterparts. Second, Deaf VGPs are anticipated to demonstrate reduced compatibility effects and be less susceptible to task-irrelevant distractors under both low and high perceptual load conditions relative to deaf NVGPs.

## 2 Methods and materials

### 2.1 Participants

To determine the appropriate sample size, we used G^★^Power software (version 3.1.9.2; Faul et al., 2009) to perform a repeated measures analysis of variance (ANOVA) with a within-between interaction factor. Input parameters included: effect size f:0.25, alpha error: 0.05, power: 0.95, number of groups: 4, number of measurements: 6, correlation among repeated measures: 0.5, and nonsphericity correction: 1. The minimum sample size was calculated to be 40^1^. To ensure high statistical power and considering the practical conditions of participant recruitment, the final sample size for this study was 115 participants, which fully meets the statistical requirements. A total of fifty-seven deaf students and fifty-eight hearing students were separately recruited from two schools in Wuhan, China: a middle school for the deaf and a neighboring vocational middle school. Participants completed a video game questionnaire as part of the screening process. They were subsequently categorized into four groups: deaf VGPs (15 female, *M*_age_ = 16.42 ± 1.27), deaf NVGPs (16 female, *M*_age_ = 16.19 ± 1.22), hearing VGPs (15 female, *M*_age_ = 16.13 ± 1.02), and hearing NVGPs (15 female, *M*_age_ = 16.07 ± 0.87).

The inclusion criteria were as follows:(a)Deaf participants had experienced profound deafness from an early age, either congenitally or before the age of three, with an average hearing loss exceeding 80 dB in both ears. None of these students used hearing aids or cochlear implants. Most began learning Chinese Sign Language (CSL) in preschool and primarily communicated through CSL. Two of the participants were proficient in both sign and spoken language. In contrast, none of the hearing participants used sign language. (b)Classification as VGPs or NVGPs followed Bavelier’s criteria (Green and Bavelier, 2003, 2006b). Specifically, VGPs were defined as individuals who reported playing action video games for at least 1 h per day on 3–4 days per week during the past six months, whereas NVGPs reported rarely or never engaging in action video game play during this period. (c) All participants had normal intelligence and normal or corrected-to-normal vision. Handedness was defined functionally, based on self-report and observation of predominant right-hand responses during the task. The Raven’s Standard Progressive Matrices (SPM) (Raven, 1941) were completed by all participants. Demographic characteristics did not differ significantly among the four groups. A chi-square test indicated no significant differences in gender distribution, χ^2^(3) = 0.589, *p* = 0.899, Cramer’s *V* = 0.072. A one-way ANOVA revealed no significant between-group differences in age, *F*(3, 111) = 0.509, *p* = 0.677, η_p_^2^ = 0.014, or in SPM scores, *F*(3, 111) = 1.558, *p* = 0.204, *η_p_^2^* = 0.04. Complete demographic information was provided in Table 1.

**Table 1:** Descriptive characteristics of the participants.

Groups	*n*	Gender (Male/Female)/*n*	Average age	SPM scores
Deaf VGPs	26	11/15	16.42 (1.27)	47.88 (7.32)
Deaf NVGPs	31	15/16	16.19 (1.22)	46.00 (8.38)
Hearing VGPs	31	16/15	16.13 (1.02)	49.16 (3.44)
Hearing NVGPs	27	12/15	16.07 (0.87)	48.67 (3.94)

The values of average age and SPM scores in the table are M (SD). A total of 118 participants were initially recruited. Three deaf participants (two deaf VGPs and one deaf NVGP) were excluded because each had more than 15 missing fixation points in the eye-tracking data. Consequently, all analyses of accuracy, reaction time, and eye-tracking measures were conducted with the final sample of 115 participants.

The questionnaire was adapted from Anderson’s Video Game Questionnaire (VGQ) (Anderson and Dill, 2000), achieving a Cronbach’s alpha coefficient of 0.84. It comprised four sections. The first section collected basic demographic information about the participants, including name, gender, age, and dominant hand. Moreover, deaf participants were asked to provide additional details such as the degree of hearing loss, age of onset, and duration of sign language use. The second section employed a five-point Likert scale to evaluate the frequency of video game usage over the past six months, with the following response options: “never played,” “played once or twice,” “rarely played,” “played occasionally,” and “played frequently.” In the third section, participants were instructed to list the three games they had played most frequently during the past six months. Finally, in the fourth section, participants used a seven-point Likert scale to rate how frequently they played each of the three games over the past six months, providing specific details such as the number of days per week and the average duration of each gaming session. Based on the questionnaire results, participants were screened and categorized according to the criteria described earlier, and were divided into two groups: action video game players and non-players. Notably, the action video games included in this study consisted of first- or third-person shooting games, such as Crossfire, Peace Elite, Crisis Action 2, Frontline Combat, and Call of Duty.

The study was approved by the Medical Ethics Committee of Wuhan Sports University (No. 2023054). Written Informed consent was obtained from the parents or guardians of all participants involved in the study.

### 2.2 Apparatus and materials

In this study, we employed the Eyelink Portable Duo eye-tracking system for data collection, which operates at a sampling rate of 2,000 Hz. The experimental stimuli were presented on a 19-inch DELL monitor with a refresh rate of 60 Hz and a resolution of 1,280 × 1,024 pixels. Participants’ eyes were positioned approximately 60 cm from the display screen.

**Figure 1:**
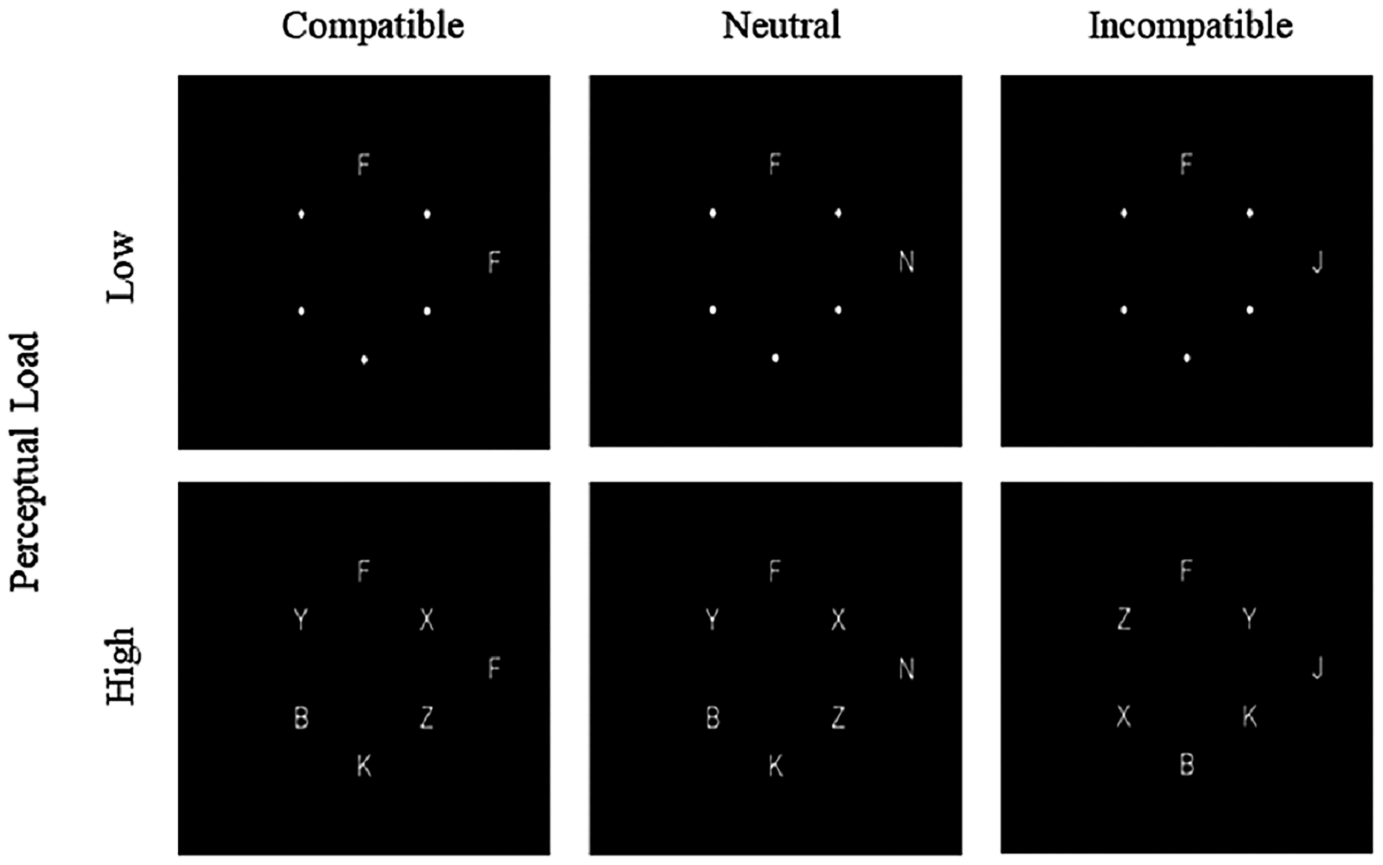
Example of stimulus material pictures.

To examine the attentional processing characteristics and between-group differences when participants were presented with peripheral distractors of different types, we developed the experimental materials based on the response competition paradigm proposed by Lavie and Cox (1997). The materials comprised 72 circular stimuli. Each stimulus contained a variable number of English uppercase letters (see Figure 1). The stimuli were presented as white letters on a black background, with each letter measuring 0.48° in both height and width. The six locations were arranged in an imaginary circle with a radius of 4.76° of visual angle from the central fixation point, with the target letter (either F or J) randomly presented at one of the six positions. The remaining five positions were occupied by nontarget letters (B, K, X, Y, Z) or small white dots. A distractor letter was always displayed at a fixed location on the right side of the screen, positioned 10° from the central fixation point. Both target and nontarget stimuli were randomly and evenly distributed across the six positions. To manipulate perceptual load, the number of nontarget letters within each circle was systematically controlled. In the low perceptual load condition, the circle contained only the target letter and five small white dots. Conversely, in the high perceptual load condition, the circle included the target letter along with five nontarget letters. The distractor letter fell into one of three categories:(a)compatible distractor, in which the distractor letter was the same as the target (e.g., F shown when the target was F);(b)neutral distractor, in which the distractor letter was an unrelated letter (N); and (c)incompatible distractor, in which the distractor letter differed from and conflicted with the target (e.g., J shown when the target was F, and vice versa).

### 2.3 Procedure

The experiment adhered to the procedures outlined as follows:

#### 2.3.1 Experiment preparation

The experimenter provided a detailed explanation of the materials and key roles to all participants. Notably, one experimenter was proficient in Chinese sign language, which used sign language and paper-and-pen communication to facilitate deaf students’ comprehension of the experimental procedures and ensured their full understanding of the experimental process.

#### 2.3.2 Calibration

Before the experiment, a standard 9-point calibration procedure was performed. Participants rested their jaws on a U-shaped mandibular tray to stabilize head position and were instructed to minimize head movements throughout the session.

#### 2.3.3 Practice trials

The experimenter delivered clear instructions to the participants, emphasizing the need to disregard distractor letters outside the circle while responding to target stimuli. Participants completed 12 practice trials to confirm their understanding of the experimental procedure before proceeding to the formal experiment.

#### 2.3.4 Formal experiment

Participants faced the computer screen, and following the completion of the calibration, they viewed the instructions displayed on the screen. They initiated the experiment by pressing the keys according to the instructions. Each trial began with the presentation of a 1,000 ms fixation cross(+) at the center of the screen. Subsequently, the central search array and distractor letter were presented for 100 ms to prevent participants from using eye movements during the visual search. Participants were required to select the target letter (either “F” or “J”) and respond by pressing the corresponding key within 3,000 ms. In cases where a participant failed to respond within the allotted time, the experiment automatically advanced to the next trial.

The formal experiment consisted of a total of 144 trials, including 24 trials for each of the three types of distractors under two distinct perceptual load conditions, as well as 12 trials for the practice phase. All experiments were conducted in a quiet room with dim lighting to minimize external interference.

### 2.4 Statistical analysis

Given that fixation-related parameters of eye movements serve as reliable indicators of perceptual load (Liu et al., 2022; Harris et al., 2023), the present study selected fixation duration and total fixation count as key eye-tracking metrics to examine the effects of two plasticity factors: action video game experience and deafness, on fixation behavior at different levels of perceptual load. Reaction time and eye movement indices were calculated exclusively from trials with correct responses. Additionally, trials exceeding three standard deviations from individuals’ mean values on these measures were excluded. Based on the exclusion criteria, a total of 14,577 valid trials remained, representing 88.03% of the overall dataset. Under the low perceptual load condition, 2,595 compatible trials (deaf VGPs = 580, deaf NVGPs = 693, hearing VGPs = 716, hearing NVGPs = 606), 2,584 neutral trials (569, 681, 717, 617), and 2,278 incompatible trials (510, 522, 680, 566) were retained. Under the high perceptual load condition, 2,519 compatible trials (554, 656, 705, 604), 2,494 neutral trials (534, 621, 719, 620), and 2,107 incompatible trials (454, 451, 656, 546) were retained.

The IBM SPSS Statistics (version 27) was employed for all data analyses. A three-factor repeated-measures ANOVA was conducted to investigate the effects of groups (deaf VGPs, deaf NVGPs, hearing VGPs, hearing NVGPs), perceptual load level (low vs. high), and distractors (compatible, neutral, or incompatible) on selective attention. The dependent variables comprised accuracy, reaction time, fixation duration, and total fixation counts. The analysis proceeded in several stages. First, the main effects of the three independent variables were tested, and when a main effect was significant, *post hoc* comparisons were conducted. Next, the interaction effects were analyzed, and for any significant interactions, simple effects analyses were performed to clarify their nature. To address potential violations of the sphericity assumption, Greenhouse–Geisser corrections were applied, and the adjusted degrees of freedom and *p* values were reported where necessary. All pairwise comparisons were computed using Bonferroni correction. Statistical significance was determined at *p* < 0.05, and effect size estimates (η_p_^2^) were also reported. Given the numerous tasks and analyses, the main effects of groups, perceptual load level, and distractors were reported and discussed in the main text. In addition, the interaction effects between groups and perceptual load level, between groups and distractors, and the three-way interaction among these three variables were examined in detail. The complete results of all main and interaction effects are provided in the Supplementary Material.

**Figure 2:**
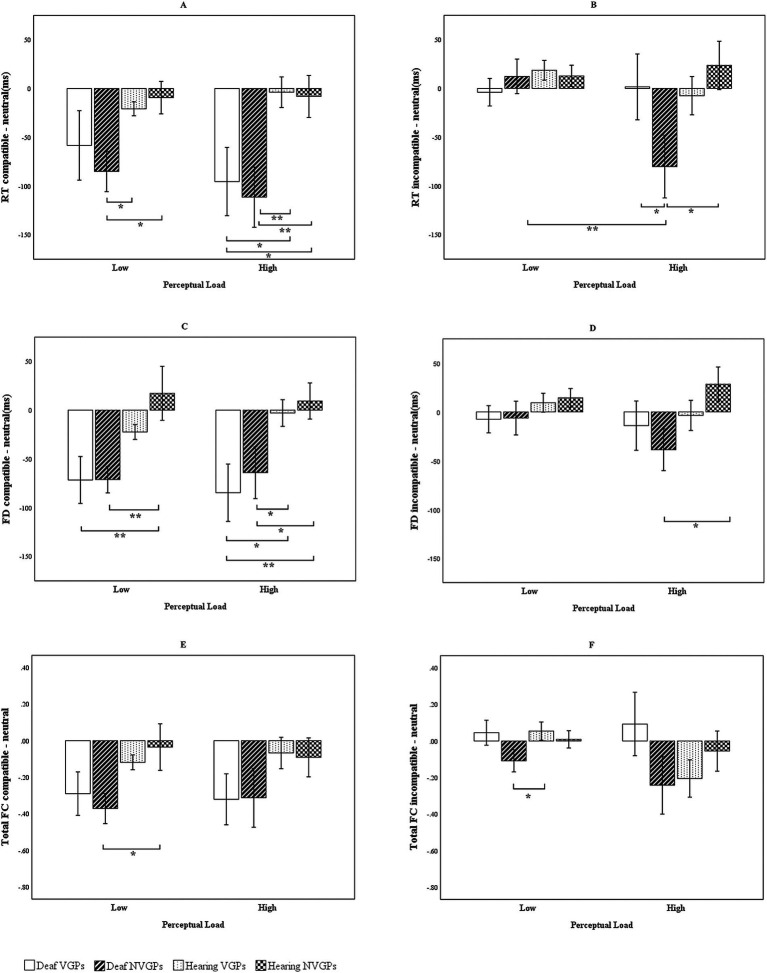
Distractor compatible effects on reaction time and eye movement indicators. **(A)** RT compatible – neutral = compatible minus neutral reaction time. **(B)** RT incompatible – neutral = incompatible minus neutral reaction time. **(C)** FD compatible – neutral = compatible minus neutral fixation duration. **(D)** FD incompatible – neutral = incompatible minus neutral fixation duration. **(E)** Total FC compatible – neutral = incompatible – neutral compatible minus neutral total fixation counts. **(F)** Total FC incompatible – neutral = incompatible minus neutral total fixation counts. The error bars represent standard errors. ^***^p < 0.001, ^**^p < 0.01, ^*^p < 0.05.

**Figure 3:**
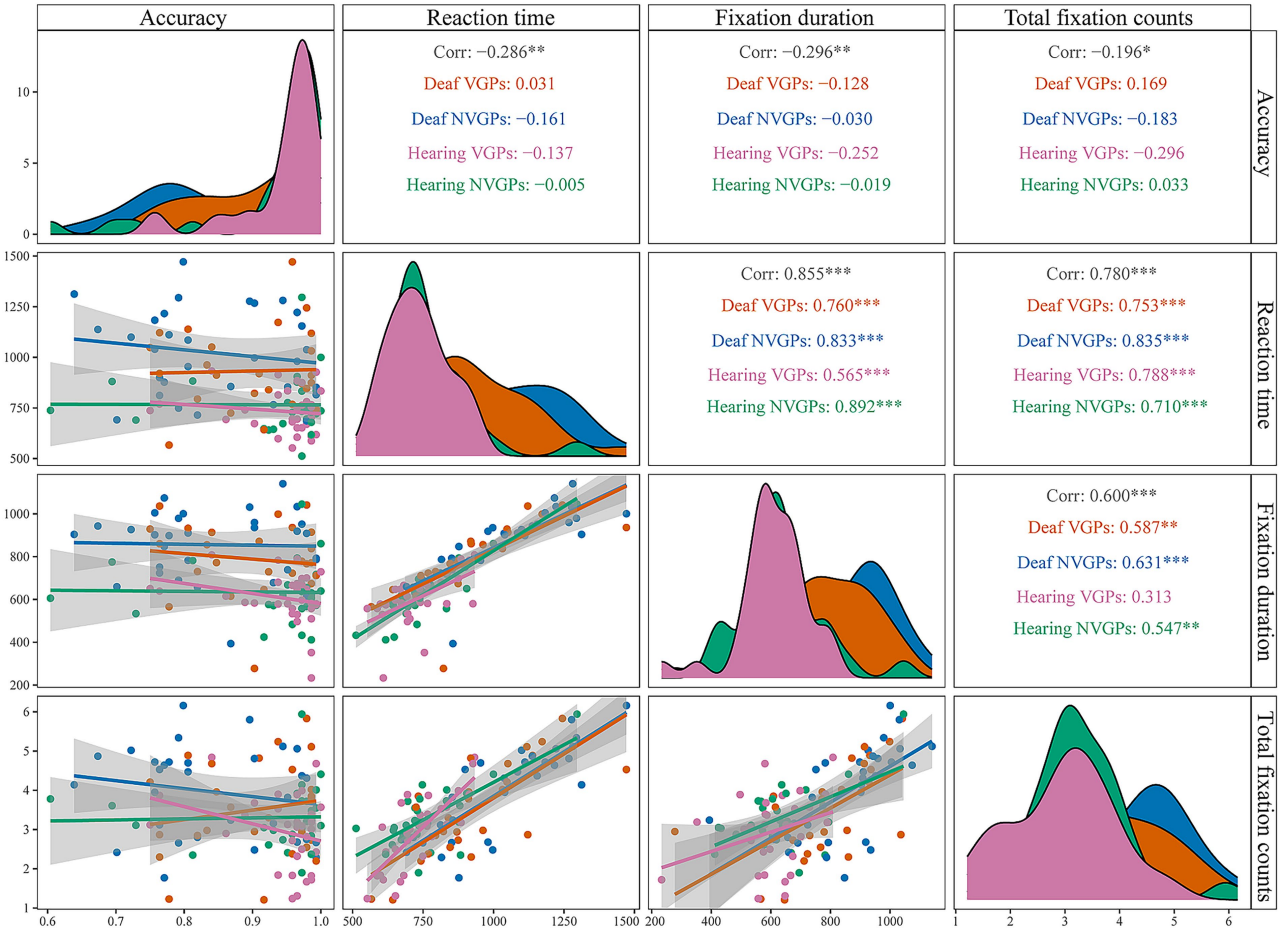
Correlation matrix between behavioral and eye-tracking measures across groups. Panels along the diagonal display the univariate distributions of the four variables (Accuracy, Reaction time, Fixation duration, and Total fixation counts). The lower-triangular panels (below the diagonal) present scatterplots with regression lines, illustrating pairwise associations between variables and subgroup patterns. The upper-triangular panels (above the diagonal) report the Pearson correlation coefficients (*r*) for all participants as well as for each group, with asterisks denoting significance levels (^**^p < 0.01, ^***^p < 0.001). Different colors represent the four groups: orange, Deaf VGPs; blue, deaf NVGPs; pink, hearing VGPs; green, hearing NVGPs.

In addition, to illustrate compatible effects, we plotted Figure 2 comparing reaction time, fixation duration, and total fixation counts by distractors. For each participant, we calculated compatible effects as the differences between the compatible and neutral distractor conditions and between the incompatible and neutral conditions for each measure, separately under low and high perceptual load. Consistent with classic studies (e.g., Lavie, 1995; Lavie and Cox, 1997), we use the neutral distractor as the baseline and quantify interference by comparing incompatible/compatible conditions against the neutral condition, which cleanly separates distinct mechanisms of distractors. These difference scores were then analyzed using a 4 (groups: deaf VGPs, deaf NVGPs, hearing VGPs, hearing NVGPs) × 2 (perceptual load level: low vs. high) repeated-measures ANOVA. Notably, accuracy is generally less sensitive to distractor interference and often approaches ceiling levels (Lavie and Cox, 1997; Maylor and Lavie, 1998; Lavie, 2005). Accordingly, Figure 2 presents reaction time results, whereas accuracy results are reported in the text with exact statistical values. To further examine whether fixation measures serve as valid indices of the effect of perceptual load on selective attention, we conducted Pearson correlation analyses between behavioral measures (mean accuracy, mean reaction time) and fixation metrics (mean fixation duration, mean total fixation count). These analyses were conducted both across all participants and within each of the four groups. In addition, we visualized the correlations by plotting the corresponding correlation matrices (Figure 3).

## 3 Results

For greater clarity, the mean values and standard deviations of accuracy, reaction time, and eye-tracking indices were provided in Table 2 for both levels of perceptual load and across the three distractor conditions.

**Table 2:** Results of behavior and eye movement indicators of participants.

Groups		Mean value	Low perceptual load			High perceptual load		
			Compatible	Neutral	Incompatible	Compatible	Neutral	Incompatible
Deaf	VGPs (*n* = 26)							
	Accuracy	0.90 (0.84)	0.95 (0.05)	0.95 (0.06)	0.86 (0.16)	0.92 (0.10)	0.93 (0.07)	0.80 (0.23)
	Reaction time	932.52 (201.60)	720.98 (210.68)	779.27 (226.55)	775.42 (223.93)	1071.17 (219.65)	1166.49 (305.97)	1168.10 (298.39)
	Fixation duration	788.35 (168.13)	616.58 (137.01)	688.12 (186.31)	680.65 (201.53)	886.70 (193.40)	971.30 (254.35)	957.25 (255.82)
	Total fixation counts	3.50 (1.21)	2.66 (0.91)	2.95 (1.10)	3.00 (1.30)	3.96 (1.41)	4.28 (1.75)	4.38 (1.75)
	NVGPs (*n* = 31)							
	Accuracy	0.84 (0.10)	0.94 (0.07)	0.93 (0.07)	0.71 (0.25)	0.92 (0.10)	0.90 (0.12)	0.66 (0.28)
	Reaction time	1022.39 (214.29) (295.65)	777.05 (202.60)	862.02 (230.49)	874.44 (259.18)	1186.89 (252.32)	1298.22 (276.14)	1218.14 (286.39)
	Fixation duration	855.96 (160.31)	656.93 (134.96)	728.08 (164.42)	721.84 (180.73)	1003.36 (205.05)	1067.42 (200.63)	1028.68 (229.44)
	Total fixation counts	3.95 (1.16)	2.93 (0.91)	3.30 (1.17)	3.19 (1.11)	4.75 (1.45)	5.06 (1.58)	4.82 (1.63)
Hearing	VGPs (*n* = 31)							
	Accuracy	0.94 (0.06)	0.96 (0.04)	0.97 (0.04)	0.92 (0.12)	0.96 (0.05)	0.97 (0.05)	0.89 (0.17)
	Reaction time	734.14 (105.33)	564.52 (82.69)	585.24 (100.45)	603.90 (104.22)	885.57 (147.94)	889.27 (164.51)	881.88 (133.30)
	Fixation duration	607.37 (116.64)	475.38 (93.74)	497.71 (109.15)	507.20 (110.74)	722.25 (147.03)	725.14 (158.93)	721.61 (152.85)
	Total fixation counts	2.96 (0.92)	2.22 (0.68)	2.34 (0.71)	2.39 (0.74)	3.63 (1.22)	3.69 (1.36)	3.49 (1.16)
	NVGPs (*n* = 27)							
	Accuracy	0.93 (0.10)	0.96 (0.06)	0.95 (0.06)	0.87 (0.20)	0.96 (0.05)	0.97 (0.04)	0.87 (0.25)
	Reaction time	765.84 (148.32)	605.04 (146.69)	614.31 (115.43)	627.24 (123.85)	910.43 (197.69)	918.48 (218.40)	942.17 (219.33)
	Fixation duration	634.43 (137.38)	525.81 (180.94)	508.49 (104.26)	523.01 (105.99)	752.45 (183.53)	743.02 (180.36)	771.46 (182.42)
	Total fixation counts	3.31 (0.80)	2.57 (0.92)	2.60 (0.52)	2.61 (0.56)	4.01 (1.24)	4.10 (1.06)	4.05 (1.22)

All specific values in the table are M (SD).

### 3.1 Accuracy

The main effect of groups was significant, *F*(3,111) = 7.670, *p* < 0.001, η_p_^2^ = 0.172. Deaf NVGPs demonstrated significantly lower accuracy than both deaf VGPs, *t*(111) = −2.429, *p* = 0.017, *d* = −0.604, and hearing NVGPs, *t*(111) = −3.737, *p* < 0.001, *d* = −0.854. However, no significant accuracy differences were found either between deaf VGPs and hearing VGPs, *t*(111) = −1.805, *p* = 0.074, *d* = −0.588, or between hearing VGPs and hearing NVGPs, *t*(111) = 0.541, *p* = 0.590, *d* = 0.154. In addition, a significant main effect of perceptual load level was also found, *F*(1, 111) = 10.864, *p* = 0.001, η_p_^2^ = 0.089. Participants achieved higher accuracy under low perceptual load than under high load. The main effect of distractors reached significance as well, *F*(1.07, 119.10) = 47.061, *p* < 0.001, η_p_^2^ = 0.298. Accuracy was significantly lower in the incompatible distractor condition than in both the compatible condition, *t*(114) = −6.560, *p* < 0.001, *d* = −0.612, and the neutral condition, *t*(114) = −6.935, *p* < 0.001, *d* = −0.647(see Table 2).

Moreover, the interaction between perceptual load level and groups was significant, *F*(3, 111) = 3.105, *p* = 0.029, η_p_^2^ = 0.077. Under low perceptual load, deaf VGPs showed accuracy comparable to hearing VGPs, *t*(55) = −1.754, *p* = 0.172, *d* = −0.480, whereas under high perceptual load, deaf VGPs were less accurate than hearing VGPs, *t*(55) = −2.259, *p* = 0.045, *d* = −0.614. Across both load levels, deaf NVGPs were consistently less accurate than the other three groups, −4.352 ≤ *ts*(51 to 60) ≤ −1.905, *ps* < 0.05, −1.105 ≤ *ds* ≤ − 0.507. In contrast, the two hearing groups showed comparable accuracy across both low load, *t*(56) = 0.941, *p* = 0.385, *d* = 0.248, and high load, *t*(56) = 0.258, *p* = 0.816, *d* = 0.068 (see Table 2). Similarly, a significant interaction was found between distractors and groups, *F*(3.22, 119.10) = 4.768, *p* = 0.003, η_p_^2^ = 0.114. Under the compatible distractor condition, deaf NVGPs exhibited lower accuracy than hearing NVGPs, *t*(56) = −1.984, *p* = 0.041, *d* = −0.512. Under the neutral distractor condition, deaf NVGPs again showed lower accuracy than hearing NVGPs, *t*(56) = −2.658, *p* = 0.004, *d* = −0.676, and deaf VGPs also performed reduced accuracy than hearing VGPs, *t*(55) = −2.450, *p* = 0.049, *d* = −0.652. Under the incompatible distractor condition, deaf NVGPs were consistently less accurate than deaf VGPs, *t*(55) = −2.409, *p* = 0.011, *d* = −0.623, and hearing NVGPs,*t*(56) = −2.920, *p* < 0.001, *d* = −0.761. Finally, the two hearing groups exhibited comparable accuracy across all distractor conditions, −0.156 ≤ *ts*(56) ≤ 0.6 70, *ps >* 0.05, −0.041 ≤ *ds* ≤ 0.176(see Table 2).

### 3.2 Reaction time

The main effect of groups was statistically significant, *F*(3, 111) = 18.363, *p* < 0.001, η_p_^2^ = 0.332. Deaf students showed longer reaction times compared to their hearing counterparts, 3.526 ≤ *ts*(111) ≤ 6.476, *ps* < 0.001, 1.005 ≤ *ds* ≤ 1.826. The reaction times of deaf VGPs were shorter than those of deaf NVGPs, *t*(111) = −1.833, *p* = 0.069, *d* = −0.428, whereas no significant difference was observed between hearing VGPs and hearing NVGPs, *t*(111) = −0.717, *p* = 0.475, *d* = −0.272. The main effect of perceptual load was also significant, *F*(1, 111) = 834.624, *p* < 0.001, η_p_^2^ = 0.883, with reaction times being shorter under low perceptual load than under high load. The main effect of distractors was significant as well, *F*(1.83, 202.73) = 15.692, *p* < 0.001, η_p_^2^ = 0.124. Participants responded faster in the compatible distractor condition than in the neutral condition, *t*(114) = −4.503, *p* < 0.001, *d* = −0.422, and the incompatible condition, *t*(114) = −4.159, *p* < 0.001, *d* = −0.388, while no significant difference was observed between the incompatible and neutral distractor conditions, *t*(114) = −0.702, *p* = 0.737, *d* = −0.065. Furthermore, a significant interaction between perceptual load and groups was observed, *F*(3, 111) = 4.181, *p* = 0.008, η_p_^2^ = 0.102. The interaction between distractors and groups also reached significance, *F*(5.48, 202.73) = 2.951, *p* = 0.011, η_p_^2^ = 0.074. Additionally, a significant three-way interaction among perceptual load, distractors, and groups was found, *F*(5.54, 205.00) = 2.605, *p* = 0.022, η_p_^2^ = 0.066, see Table 2.

This three-way interaction in the reaction time data was illustrated in Figures 2A,B. These figures depict the differences in reaction times between compatible and neutral distractors, as well as between incompatible and neutral distractors, across low and high perceptual load conditions, reflecting the magnitude of the compatible effects. Under low perceptual load, the compatible effect observed in deaf NVGPs was larger than that of hearing NVGPs, *t*(56) = −2.851, *p* = 0.014, *d* = −0.736. Under high perceptual load, the compatible effect among deaf participants—particularly deaf NVGPs—increased further and was significantly greater than that of the hearing groups, −3.109 ≤ *ts*(51 to 60) ≤ −2.122, *ps* < 0.05, −0.790 ≤ *ds* ≤ − 0.588 (Figure 2A). Moreover, as perceptual load increased, deaf NVGPs demonstrated a larger compatible effect (incompatible-neutral) compared to deaf VGPs, *t*(55) = −1.745, *p* = 0.043, *d* = −0.464, and hearing NVGPs, *t*(56) = −2.498, *p* = 0.01, *d* = −0.658 (Figure 2B). For deaf NVGPs, the compatible effect under high load was significantly greater than under low load, *t*(30) = −2.504, *p* = 0.002, *d* = −0.450, whereas the compatible effects for other three groups did not differ significantly across perceptual load levels, −1.162 ≤ *ts*(25 to 30) ≤ −0.176, *ps* > 0.05, −0.209 ≤ *ds* ≤ − 0.035 (Figure 2B).

### 3.3 Fixation duration

The main effect of groups was statistically significant, *F*(3, 111) = 19.753, *p* < 0.001, η_p_^2^ = 0.348. Deaf VGPs and NVGPs showed longer fixation durations than their hearing counterparts, 3.844 ≤ *ts*(111) ≤ 6.631, *ps* < 0.001, 1.084 ≤ *ds* ≤ 1.851, whereas no significant difference was found between deaf VGPs and deaf NVGPs, *t*(111) = −1.650, *p* = 0.102, *d* = −0.413, nor between hearing VGPs and hearing NVGPs, *t*(111) = −0.719, *p* = 0.474, *d* = −0.230. The main effect of perceptual load level was also significant, *F*(1, 111) = 801.007, *p* < 0.001, η_p_^2^ = 0.878, with fixation durations being shorter under low perceptual load than under high load. The main effect of distractors was significant as well, *F*(1.72, 190.86) = 11.762, *p* < 0.001, η_p_^2^ = 0.096. Fixation durations were shorter in the compatible condition than in the neutral condition, *t*(114) = −3.808, *p* < 0.001, *d* = −0.355, and the incompatible condition, *t*(114) = −3.247, *p* < 0.001, *d* = −0.303, while no significant difference was found between the incompatible and neutral conditions, *t*(114) = −0.892, *p* = 0.735, *d* = −0.083. In addition, a significant interaction was observed between perceptual load and groups, *F*(3, 111) = 6.384, *p* < 0.001, η_p_^2^ = 0.147. The interaction between distractors and groups was also significant, *F*(5.16, 190.86) = 3.488, *p* = 0.004, η_p_^2^ = 0.086, see Table 2.

Figures 2C,D illustrate the compatible effects on fixation duration, showing patterns similar to those observed in reaction time. Specifically, deaf students exhibited larger compatible effects than hearing students under both perceptual load conditions, with the difference particularly marked under high load, −2.704 ≤ *ts*(51 to 60) ≤ −2.263, *ps* < 0.05, −0.749 ≤ *ds* ≤ − 0.579 (see Figure 2C). Deaf NVGPs showed greater compatible effects than hearing NVGPs under high load conditions, *t*(56) = −2.375, *p* = 0.019, *d* = −0.625 (Figure 2D). In contrast, the compatible effects for hearing VGPs and NVGPs were not significant under either level of perceptual load, −1.464 ≤ *ts*(56) ≤ −0.369, *ps >* 0.05, −0.385 ≤ *ds* ≤ − 0.097 (Figures 2C,D).

### 3.4 Total fixation counts

The main effect of groups was significant, *F*(3, 111) = 5.033, *p* = 0.003, η_p_^2^ = 0.120. Deaf NVGPs exhibited more fixations than hearing NVGPs, *t*(111) = 3.809, *p* = 0.018, *d* = 1.040, and deaf VGPs also showed more fixations than hearing VGPs, *t*(111) = 2.003, *p* = 0.048, *d* = 0.544. In contrast, no significant differences were found between deaf VGPs and deaf NVGPs, *t*(111) = −1.635, *p* = 0.105, *d* = −0.399, or between hearing VGPs and hearing NVGPs, *t*(111) = −1.274, *p* = 0.205, *d* = −0.419. The main effect of perceptual load level was statistically significant, *F*(1, 111) = 469.411, *p* < 0.001, η_p_^2^ = 0.809, with fewer fixations under low load than under high load. The main effect of distractors was also significant, *F*(1.87, 207.84) = 10.269, *p* < 0.001,η_p_^2^ = 0.085. Total fixation counts were lower for compatible distractors than for incompatible distractors, *t*(114) = −2.449, *p* = 0.004, *d* = −0.228, and neutral distractors, *t*(114) = −4.235, *p* < 0.001, *d* = −0.395, while no significant difference was found between the neutral and incompatible conditions, *t*(114) = 1.862, *p* = 0.205, *d* = 0.174. No significant interaction was observed between perceptual load and groups, *F*(3, 111) = 2.431, *p* = 0.069, η_p_^2^ = 0.062. The interaction between distractors and groups was significant, *F*(5.62, 207.84) = 2.214, *p* = 0.047, η_p_^2^ = 0.056, see Table 2.

Figures 2E,F illustrate the compatible effects on total fixation counts, although the effects appeared less substantial than those observed in reaction time and fixation duration. Specifically, under low perceptual load conditions, deaf NVGPs demonstrated greater compatible effects than hearing students, −2.269 ≤ *ts*(51 to 60) ≤ −2.074, *ps* < 0.05, −0.597 ≤ *ds* ≤ − 0.527 (see Figures 2E,F). As perceptual load increased, the compatible effects also increased across all four groups (Figure 2F).

In summary, Figure 2 demonstrated that the compatible effects observed in the deaf groups were larger than those in the hearing groups. Deaf students exhibited clear compatible effects under both low and high levels of perceptual load, with effects being especially greater under high load, particularly among deaf NVGPs. In contrast, hearing students did not show an evident compatible effect under either load level. Furthermore, eye movement measures, including total fixation counts and especially fixation duration, reflected similar patterns of distractor compatible effects as those found in reaction time data.

### 3.5 Correlation analysis between behavioral indicators and fixation-related parameters

For all participants, the results showed a negative correlation between accuracy and fixation duration, *r*(113) = −0.296, *p* = 0.001, as well as between accuracy and total fixation counts, *r*(113) = −0.196, *p* = 0.036. Conversely, reaction time was positively correlated with fixation duration, *r*(113) = 0.855, *p* < 0.001, and with total fixation counts, *r*(113) = 0.780, *p* < 0.001 (see Figure 3). For each group, a consistent pattern was observed (see Figure 3): In all four groups, accuracy was not significantly correlated with fixation duration or total fixation counts (*ps* > 0.05), whereas reaction time was positively correlated with both measures (*ps* ≤ 0.001). Representative coefficients were relatively large; for example, reaction time was positively correlated with fixation duration in deaf NVGPs, *r*(29) = 0.833, *p* < 0.001, and in hearing NVGPs, *r*(29) = 0.892, *p* < 0.001. Detailed coefficients for each group are provided in Figure 3.

## 4 Discussion

### 4.1 Differences in selective attention between deaf and hearing students

The behavioral indicators of the present study revealed that deaf students exhibited generally poorer performance compared to their hearing counterparts in terms of accuracy and reaction time during selective attention tasks. This result is consistent with prior research, which indicates that inadequate auditory input contributes to substantial deficits in visual selective attention among the deaf population (Quittner et al., 1994; Gioiosa Maurno et al., 2024). Early auditory deprivation can lead to challenges in multisensory integration and the endogenous regulation of visual attentional resources, thereby limiting the capacity of deaf individuals to execute specific tasks and monitor their surrounding environment effectively. Such limitations may result in heightened impulsivity and difficulties in sustaining focused attention (Smith et al., 1998; Quittner et al., 2004). Intriguingly, we found that the compatible effect was markedly greater in deaf students than in their hearing peers, which aligns with previous studies (Proksch and Bavelier, 2002; Dye et al., 2009; Chen et al., 2010). These studies generally propose that deaf individuals process peripheral visual information in a manner that diminishes the availability of resources for central visual processing. Consequently, they reallocate their visual attentional resources toward peripheral spaces, enhancing attentional selection for peripheral vision relative to central vision. Nevertheless, some studies have reported no significant difference in the ability to process peripheral distractors between deaf and hearing populations (Holmer et al., 2020; Daza González et al., 2021). The eye movement metrics of this study demonstrated that deaf students had longer fixation durations, higher total fixation counts, and larger compatible effects compared to hearing students. These findings suggest that a larger proportion of their attentional resources was allocated to the peripheral visual field. The influence of deafness on visual attention is evident not only in the distribution of attentional resources but also in oculomotor control mechanisms.

Different levels of perceptual load exert a substantial influence on selective attention in deaf students. The perceptual load inherent to a task dictates the allocation of attentional resources. At low perceptual loads, surplus resources are automatically allocated, potentially leading to the processing of distracting stimuli. Conversely, at high perceptual loads, attention is entirely consumed by the task, and distractors are generally not processed (Lavie, 2005). Our study revealed that perceptual load significantly impacted accuracy, reaction time, and eye movement metrics, including fixation duration and total fixation counts among deaf students. However, our results demonstrated a significant compatibility effect under high perceptual load conditions compared to low perceptual load conditions. This finding diverges from the research by Lavie and Cox (1997), which demonstrated significant compatibility effects under low perceptual loads but none under high perceptual loads. The discrepancy in results may stem from the different visual attention characteristics of deaf individuals, who tend to redistribute their attentional resources from the central visual field to the periphery. This redistribution leads to a greater allocation of resources to peripheral distractors, thereby amplifying the compatibility effect under high perceptual load. This research underscores the distinctive pattern of attentional distribution in deaf students and its implications for the compatible effect. Furthermore, across all participants, we observed significant correlations between eye movement metrics (including fixation duration and total fixation counts) and behavioral measures (accuracy, particularly reaction time). As the perceptual load increased, both fixation duration and fixation counts increased for all participants. This result is consistent with the findings of Harris et al. (2023). These findings suggest that these two metrics serve as sensitive indicators of perceptual load changes and may be reliably used as gaze metrics for assessing the perceptual load. Notably, although we found negative correlations between accuracy and fixation-related parameters across all participants, no such associations were observed within any of the four groups. This further supports the findings of Lavie (1995), which showed that, relative to reaction time, accuracy was a less sensitive measure of perceptual load.

### 4.2 Effects of action video game experience on selective attention in deaf students

The behavioral indicators of the present study suggest the following: First, deaf VGPs demonstrated better accuracy and shorter reaction time than deaf NVGPs on selective attention tasks, and their accuracy was comparable to that of hearing participants. Action video games have been shown to enhance players’ visual selective attention by improving cognitive functions such as perception, spatial awareness, top-down attentional control, task switching, and cognitive flexibility (Bediou et al., 2018; Föcker et al., 2018). Additionally, Holmer et al. (2020) reported that deaf gamers outperformed their deaf non-gamer counterparts in visuospatial attention tasks. Nagendra et al. (2017) demonstrated that a video game intervention improved the attentional capabilities of deaf students. Furthermore, research has demonstrated that both central and peripheral visual fields were found to be larger in VGPs and deaf individuals compared to hearing controls (Buckley et al., 2010). Our study confirmed that Deaf VGPs outperformed Deaf NVGPs in selective attention tasks, although they still performed inferior to hearing controls. In the research by Holmer et al. (2020), deaf gamers performed comparably to hearing individuals on a visuospatial attention task, whereas deaf non-gamers struggled. The inconsistencies between our findings and those of this study may stem from variations in the definitions of gamer criteria or the experimental tasks employed. Second, eye movement metrics in the present study revealed no significant differences in fixation duration or total fixation counts between deaf VGPs and deaf NVGPs. This result is consistent with the ongoing debate in the current research domain (Montolio-Vila et al., 2024). Several studies support the eye-tracking findings reported here. For instance, Azizi et al. (2017) found no significant differences in fixation duration between VGPs and NVGPs, a finding confirmed by both cross-sectional comparisons and a 10-h training intervention. Similarly, Peters et al. (2021) and Delmas et al. (2022) reported no substantial differences in fixation-related metrics. However, other studies have presented contrasting evidence. West et al. (2013), Jeong et al. (2022), and Li et al. (2022) found that VGPs exhibited certain advantages, such as shorter fixation duration and fewer fixations. While there is substantial evidence that action video game experience can enhance attentional performance, its impact on eye movement behavior appears to be more limited.

We also found that, as the perceptual load increased, the distractor compatibility effect was markedly diminished in Deaf VGPs compared to Deaf NVGPs. Previous studies have demonstrated that both VGPs and NVGPs exhibit a compatibility effect under conditions of low perceptual load. However, under high perceptual load, only VGPs manifest a compatible effect (Green and Bavelier, 2003, 2006a). Our study revealed that both Deaf VGPs and Deaf NVGPs exhibited distractor compatibility effects under low perceptual load. In contrast, under high perceptual load, deaf NVGPs displayed more significant compatibility effects than other groups. The discrepancies in these findings may be attributed to the synergistic influence of auditory deprivation and action video game experience among deaf individuals. Early auditory deprivation redirects visual attentional resources from the central to the peripheral visual field in deaf individuals, impairing information processing in the foveal region while enhancing attentional allocation to peripheral areas, thereby augmenting peripheral visual attention (Dye, 2016). Moreover, experience with action video games alters the spatial distribution of visual attentional resources in deaf individuals, enhancing their resistance to peripheral distractors and improving selective attention efficiency.

### 4.3 Limitations

Our study acknowledges several limitations that warrant consideration. Firstly, due to practical constraints, we were unable to assess language proficiency in participants, a factor that might have contributed to the observed selective attention differences between deaf and hearing students (Dye and Terhune-Cotter, 2023). Future research is warranted to systematically examine the language proficiency and acquisition histories of deaf participants in order to clarify the role of auditory experience in shaping attentional processes. Secondly, the variety of action video games played by deaf students suggests that different game genres may exert distinct influences on cognitive enhancement. Future research should systematically investigate the effects of specific game types on the visual attention of deaf students. Thirdly, we note that we did not administer a standardized handedness inventory or assess retraining history. Consequently, innate right-handedness cannot be confirmed for all participants, which may introduce measurement noise and limit generalizability. Future studies will include a validated handedness measure and an explicit retraining-history item to better characterize handedness in the Chinese cultural context. Fourthly, Latin letters were used as distractors in this study. Although a neutral baseline condition was included, we did not directly assess Chinese participants’ familiarity with Latin letters, which may have influenced recognition of different distractor types. Future studies could consider using pictorial distractors instead to better examine the role of distractors. Additionally, investigating the selective attention characteristics of individuals with and without experience in action video games, without incorporating an experimental intervention, limits the ability to establish a causal relationship between action video games and visual selective attention. Future studies should consider integrating action video game training programs to address this methodological limitation. Lastly, another limitation of the present study concerns the *a priori* power analysis. As noted earlier in the manuscript, we incorrectly used G^★^Power to estimate the required sample size before data collection. Therefore, the originally reported estimates are only approximate and should be interpreted with caution. We did not perform a replacement calculation using more appropriate software. Future research should employ tools capable of modeling higher-order interactions and mixed-effects designs (e.g., PANGEA) and pre-register the power analysis in accordance with the finalized design.

## 5 Conclusion

In conclusion, the present study identified key features of selective attention processing in deaf students and examined the factors that influence these processes. Compared to their hearing counterparts, deaf students demonstrated generally lower selective attention performance and less efficient fixation behavior, manifesting as reduced accuracy, longer reaction times, prolonged fixation durations, and increased fixation counts. Importantly, while action video game experience was associated with enhanced selective attention performance in deaf students, its influence on eye movement behavior appeared to be limited, and these findings should be interpreted with caution because no video game intervention was conducted in this study. Furthermore, perceptual load significantly modulated both attentional performance and fixation patterns. Taken together, the findings suggest a potential, although not conclusive, role of action video game training as a supplementary approach to support the development of selective attention in deaf students. Cautious consideration of such training in special education contexts is warranted, particularly with attention to the moderating role of perceptual load in instructional design.

## Data Availability

The raw data supporting the conclusions of this article will be made available by the authors, without undue reservation.

## Footnotes

^1^The sample size estimation originally reported was conducted using G^★^Power. However, G^★^Power is limited to designs involving a single between-subjects factor and a single within-subjects factor and therefore cannot appropriately accommodate the 4 (groups) × 2 (perceptual load level) × 3 (distractors) mixed design employed in the present study. We acknowledge this methodological limitation.
