# Supplementary material for: The impact of action video game experience on visual selective attention in deaf middle school students
Source: Front Psychol. 2025 Oct 1;16:1633957. doi: 10.3389/fpsyg.2025.1633957 (PMC12520961; doi:10.3389/fpsyg.2025.1633957)
Supplement: Supplementary file 1 [file Data_Sheet_1.pdf]

1 Supplementary Tables

TABLE S1 SPSS results for the accuracy

Variables	*F*	*p*	η_p^2
Groups	7.670	**<0.001**	0.172
Perceptual load level	10.864	**0.001**	0.089
Distractors	47.061	**<0.001**	0.298
Perceptual load level* Groups	3.105	**0.029**	0.077
Distractors* Groups	4.768	**0.003**	0.114
Perceptual load level*Distractors	5.274	**0.009**	0.045
Perceptual load level*Distractors*Groups	0.270	0.932	0.007

TABLE S2 SPSS results for the reaction time

Variables	*F*	*p*	η_p^2
Groups	18.363	**<0.001**	0.332
Perceptual load level	834.624	**<0.001**	0.883
Distractors	15.692	**<0.001**	0.124
Perceptual load level* Groups	4.181	**0.008**	0.102
Distractors* Groups	2.951	**0.011**	0.074
Perceptual load level*Distractors	1.919	0.153	0.017
Perceptual load level*Distractors*Groups	2.605	**0.022**	0.066

TABLE S3 SPSS results for the fixation duration

Variables	*F*	*p*	η_p^2
Groups	19.753	**<0.001**	0.348
Perceptual load level	801.007	**<0.001**	0.878
Distractors	11.762	**<0.001**	0.096
Perceptual load level* Groups	6.384	**<0.001**	0.147
Distractors* Groups	3.488	**0.004**	0.086
Perceptual load level*Distractors	0.615	0.542	0.006
Perceptual load level*Distractors* Groups	1.069	0.382	0.028

TABLE S4 SPSS results for total fixation counts

Variables	*F*	*p*	η_p^2
Groups	5.033	**0.003**	0.120

Perceptual load level	469.411	**<0.001**	0.809
Distractors	10.269	**<0.001**	0.085
Perceptual load level* Groups	2.431	0.069	0.062
Distractors* Groups	2.214	**0.047**	0.056
Perceptual load level*Distractors	1.789	0.170	0.016
Perceptual load level*Distractors* Groups	1.022	0.412	0.027
